# A multistep in vitro hemocompatibility testing protocol recapitulating the foreign body reaction to nanocarriers

**DOI:** 10.1007/s13346-022-01141-6

**Published:** 2022-03-22

**Authors:** Valeria Perugini, Ruth Schmid, Ýrr Mørch, Isabelle Texier, Martin Brodde, Matteo Santin

**Affiliations:** 1 Centre for Regenerative Medicine and Devices, School of Applied Sciences, University of Brighton - Huxley Building, Lewes Road, Brighton, BN2 4GJ UK; 2 Department of Biotechnology and Nanomedicine, SINTEF, Trondheim, Norway; 3 Univ. Grenoble Alpes, CEA, LETI-DTBS, 38000 Grenoble, France; 4 OxProtect GmbH, Munster, Germany

**Keywords:** Nanobiomaterials, Drug nanocarriers, Polymeric nanoparticles, Lipid nanoparticles, Hemocompatibility, In vitro tests, Host response, Protein corona, Thrombogenicity, Cytotoxicity, Inflammatory response

## Abstract

The development of drug nanocarriers based on polymeric, lipid and ceramic biomaterials has been paving the way to precision medicine, where the delivery of poorly soluble active compounds and personalized doses are made possible. However, the nano-size character of these carriers has been demonstrated to have the potential to elicit pathways of the host response different from those of the same biomaterials when engineered as larger size implants and of the drugs when administered without a carrier. Therefore, a specific regulatory framework needs to be made available that can offer robust scientific insights and provide safety data by reliable tests of these novel nano-devices. In this context, the present work presents a multistep protocol for the in vitro assessment of the hemocompatibility of nanocarriers of different physicochemical properties. Poly (ethyl butyl cyanoacrylate) nanoparticles and lipid-based (LipImage™ 815) nanoparticles of comparable hydrodynamic diameter were tested through a battery of assays using human peripheral blood samples and recapitulating the main pathways of the host response upon systemic administration; i.e., protein interactions, fibrinogen-platelet binding, cytotoxicity, and inflammatory response. The data showed the sensitivity and reproducibility of the methods adopted that were also demonstrated to determine individual variability as well as to discriminate between activation of pathways of inflammation and unintended release of inflammatory signaling caused by loss of cell integrity. Therefore, this multistep testing is proposed as a reliable protocol for nanoparticle development and emerging regulatory frameworks.

**Graphical abstract:**

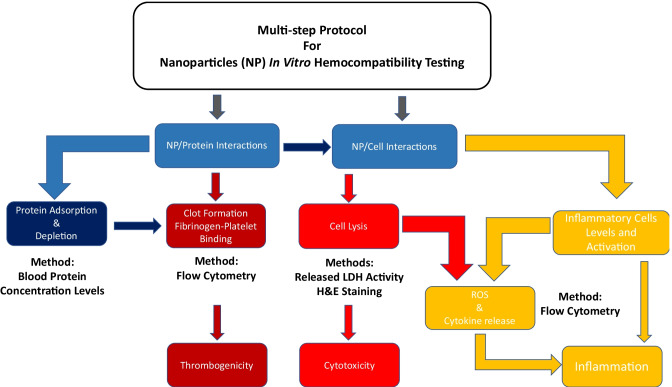

**Supplementary information:**

The online version contains supplementary material available at 10.1007/s13346-022-01141-6.

## Introduction

The clinical availability of nanocarriers for the delivery of therapeutic agents to target diseased tissues more efficaciously depends on the accurate assessment of their biocompatibility [1]. It is widely accepted that nanocarriers, typically not exceeding 200 nm, induce interactions with the biochemical and cellular components that are different from those of relatively larger particles or implants made of biomaterials bearing the same chemical moieties (e.g., hydrophobic domains and electrostatic charges) [2]. These differences can be ascribed to the nanoparticle (NP) size that increases the surface available to the adsorption and conformational changes of proteins and that are in the range favorable to their internalization by immunocompetent and tissue cells [3–5]. Indeed, rapid protein adsorption and contact with different types of cells take place soon after entering the body, regardless of the route of administration, upon contact with body fluids. In the case of systemic injection, NP will undergo an early interaction with circulating proteins and immune cells and later accumulate in tissues where a range of cells have the ability to internalize them [4, 6].

Therefore, while intravenous administration is a route that has the advantage of delivering NP systemically escaping first-pass metabolism and improving the therapeutic profile, it is also an environment where the interactions of NP with proteins and cells are enhanced [7]. In particular, through this route, the immunological response to the injected nanocarriers is increased significantly by their direct exposure to the circulating humoral and cellular elements key to the host response to a foreign body. Indeed, it is widely recognized that protein adsorption and cell contacts may activate pathways of the host response such as thrombogenicity and inflammation, which may be due either to the direct recognition of the nano-biomaterials or as a result of the unintended release of pro-inflammatory cytokines caused by cytotoxicity and consequent loss of cell integrity [8].

Therefore, the regulatory approval of novel nanocarriers depends on the successful demonstration of NP biocompatibility through a sequence of in vitro and in vivo tests that can assess any cytotoxic or immunological effect elicited upon their administration [8]. In vitro tests are also particularly important to optimize nanocarriers as they enable scientists in both academia and industry to screen a wide range of formulations before progressing the best candidates to the pre-clinical animal models. To this end, the availability of a range of relatively rapid and operator-friendly in vitro tests able to recapitulate the main biochemical and cellular pathways characterizing the host response to nano-biomaterials is advocated. In those cases where a systemic administration is pursued, hemocompatibility tests are required. Relevant to NP designed for systemic administration are the many in vitro models and assays that have been published where polymeric, lipid, metallic and ceramic nanocarriers and nanoparticles have been incubated in whole blood and tested for their thrombogenicity, immunological potential, and cytotoxicity [9–11]. While offering valuable scientific insights, these tests tend to focus on specific pathways of hemocompatibility, and they are not always designed to provide a clear overview informing scientists and regulators about the hemocompatibility of a nano-biomaterial in a manner that recapitulates the sequence of the main events triggered by its systemic administration [10]. It is also argued that the reliability of any in vitro model of hemocompatibility does not rely only on its reproducibility but also on its potential to provide data of individual variability as well as adaptability to nano-biomaterials with different physicochemical properties.

In this work, a series of interrelated tests based on incubation of either whole human blood or platelet-rich plasma (PRP) with nano-biomaterials is proposed. The combined tests aimed to recapitulate the sequence of events occurring upon systemic administration; from the early protein adsorption to the overall cytotoxicity, to thrombogenicity potential, and the release of pro-inflammatory cytokines evaluated in terms either of active release from immunocompetent cells or of passive release caused by the loss of cell integrity. The resulting multistep hemocompatibility protocol was validated by the use of nano-biomaterials with different physicochemical properties; i.e. polymeric [poly(ethyl butyl byanoacrylate), PEBCA] and lipid (LipImage™ 815) nanocarriers were selected because of their previously defined physicochemical and biocompatibility properties [12–15].

PEBCA-based NP is biodegradable drug nanocarriers that have been exhaustively characterized in vitro as well as by pre-clinical in vivo models and clinical trials [12–14]. PEBCA NP used in this study typically show a hydrodynamic diameter ranging between 100 and 200 nm with a polydispersity index ranging between 0.1 and 0.18. Their cytotoxic effects have been assessed at a concentration ranging from 0.3 to 285$\mu$g/mL within 24 h showing large variations between different cell lines and showing to be internalized by various cell lines. LipImage^TM^ 815 are lipid nanoparticles with a hydrodynamic diameter of approximately 50 nm, a polydispersity index lower than 0.25, and carrying the IR780 fluorophore [15]. Their proposed use for tumor imaging has been supported by in vitro cytotoxicity studies on fibroblast cell lines as well as by in vivo imaging determining their biodistribution [16, 17]. The assessment of the cytotoxicity showed that fibroblasts well tolerate these nanoparticles with a half-maximal inhibitory concentration being 1 mg/mL (i.e., 1.5 × 10^13^ particles/mL), while a higher accumulation in the liver than other organs was observed within 24 h. These data were considered suitable benchmarks for the validation of a multistep, operator-friendly in vitro hemocompatibility testing protocol based on the use of freshly isolated human peripheral blood. The protocol sequential steps aimed to provide interrelated information about the main checkpoints of the host response to NP without the need to undergo more convolute methods of protein adsorption and cell isolation. In addition, the use of donors’ blood made the protocol suitable to discriminate individual host response variability.

## Materials and methods

Tests were designed to study the hemocompatibility of the nanocarriers to reflect their early (minutes) and protracted (24 h) interaction with blood proteins and cells. An assay of platelet-fibrinogen binding was used as early thrombogenic potential, while cell lysis and pro-inflammatory cytokine release were measured over a longer period of time.

### Nanocarrier preparation

PEBCA and LipImage suspensions were prepared as previously reported [12]. Briefly, PEBCA nanoparticles were synthesized under aseptic conditions at SINTEF (Trondheim, Norway) by mixing an oil phase consisting of PEBCA containing 0.2% *w*/*v* Nile Red with an aqueous phase consisting of 0.1M HCl containing the two PEG stabilizers (Brij®L23 and Kolliphor®HS15). The oil in water mini-emulsion was prepared using a tip sonifier. The polymerization was then carried out overnight and unreacted monomer and surplus of surfactants were removed by extensive dialysis. Batches of LipImage^TM^ 815 were prepared by high-pressure homogenization (HPH), the lipid phase comprising soybean oil, Suppocire^TM^ NB, lecithin, and IR-870 oleyl. The aqueous phase comprised Myrj^TM^ S40 and NaCl. Mixtures of lipid and aqueous phases were pre-emulsified and then processed with a high-pressure homogenizer (Panda Plus 2000, GEA Niro Soavi, Italy) operated for 16 cycles with a total pressure of 1250 bars. NP was then purified through a 5-µm filter followed by tangential flow filtration, dispersed to a concentration of 100 mg/mL, and filter-sterilized through a 0.22-μm Millipore membrane before storage and use. Both NP types were pre-equilibrated at room temperature for 15 min and re-suspended by careful vortexing to prepare standards. These were prepared by the serial dilution of the mother solution in sterile phosphate-buffered saline (PBS) pH=7.2 at different concentrations in the range of 1 to 1000$\mu$g/mL. Samples were stored at room temperature and used within 2 h of preparation.

### Human peripheral blood collection

Human peripheral blood (4 mL) was collected from 6 healthy volunteers from both genders using the Vacutain system into heparinized tubes (BD Vacutainer^TM^ UK). In the case of platelet-fibrinogen binding experiments, blood was collected by Monovette tubes containing sodium citrate. Blood collection was authorized by the University of Brighton Ethics Committee and performed under the Human Tissue Act license no. 12583.

### Protein concentration

Protein concentration levels in donors’ blood samples incubated with different NP concentrations (6.25, 12.5, 25, 50, 100, 250, 500, and 1000 µg/mL) were performed as an indirect method to assess the early interactions between NP and blood components. Samples (50 µL) of freshly isolated human peripheral blood were added to the wells of 96-well tissue culture plates (TCP, Corning UK) and spiked with 10$\mu$L of NP at different concentrations within 2 h from phlebotomy. Each concentration was tested in duplicate for each donor. Samples were incubated in a humidified 37 °C, 5% CO_2_ cell incubator for 24 h under static conditions. After 24 h, blood was removed, and the supernatants were immediately tested for protein concentration. Protein concentrations were measured by a Bradford assay (BioRad, UK) on 5 $\mu$L of blood supernatant and data expressed as $\mu$g/mL mean ± standard deviation from *n* = 6 as calculated by a bovine serum albumin standard curve.

### Thrombogenicity

A method for the measurement of platelet-fibrinogen binding was adopted to assess the thrombogenicity potential of the tested nano-biomaterials. PRP was prepared from 5 mL of whole blood by centrifugation at 200 × *g* for 10 min. Fluorisothiocyanate (FITC)-labeled fibrinogen (OxProtec, Germany) was added to the PRP to a final concentration of 150 µg/mL, and the platelet count was adjusted to 25,000$\mu$g/µL to get the stock solution. A volume (10$\mu$l) of either NP at different concentrations or positive controls (i.e., 2 $\mu$g/mL collagen in PBS, OxProtec) were added to 100 µL of the stock solution and incubated for exactly 5 min. PBS was used as a negative control. The reaction was stopped by adding 110 µL of 1% (*v/v*) formaldehyde solution to fix the platelets for 30 min. After a washing step, the binding of FITC-labeled fibrinogen to the platelets was measured by flow cytometry by a BD Accuri C6 flow cytometer, and results were analyzed using the instrument software. Data were expressed as a percentage of the positive control.

### Cytotoxicity

Overall blood cell toxicity induced by the different concentrations of NP was assessed both in the blood supernatants and on cells deposited on the bottom of the well. The former was quantitatively determined by the release of the lactate dehydrogenase enzyme (LDH) activity, while the latter was qualitatively assessed by a hematoxylin/eosin (HE) staining method.

LDH activity was measured on aliquots obtained from the same blood samples tested for protein concentration levels. Aliquots of 50$\mu$l were tested by the CytoTox 96® non-radioactive cytotoxicity assay (Promega, UK), and data were expressed as mean percentage of the positive control ± standard deviation from *n* = 6 according to the formula:

LDH activity (% O.D. 490 nm) = [(experimental sample – background)/(positive control − background)] × 100.

where the maximum LDH release is the enzymatic activity measured in the supernatant of samples treated with 10 $\mu$L of manufacturer’s lytic solution for 45 min prior to addition of the kit substrate solution. Blood basal LDH activity and the LDH activity of blood samples treated with the lytic solution (positive control) were expressed as arbitrary units_Abs490nm_/mL.

Cells deposited at the bottom of the wells used for protein concentration and LDH activity measurements were washed twice in 50 µL of PBS, fixed by 50 µL of 3.7% *v/v* paraformaldehyde for 10 min at room temperature, washed three times with PBS, and stained by hematoxylin solution (Sigma, UK) adding 50 µL of dye into each well for 5 min at room temperature. Samples were rapidly rinsed three times with distilled water and in 0.5% *v*/*v* acetic acid for 40 s, washed again in distilled water, and counterstained in 50 µL eosin solution (Sigma, UK) for 2 min at room temperature. The samples were finally rinsed three times in water. Light microscopy images were taken from samples of the two types of nanoparticles at × 20 magnification and qualitatively analyzed for their area coverage.

### Inflammatory cell levels and response

The levels and activation of inflammatory cells involved in the early inflammatory response, granulocytes, and monocytes/macrophages, were assessed in the whole blood samples challenged by different concentrations of NP (1, 2, 5, 10, 100, and 1000${\varvec{\mu}}$g/mL) by flow cytometry. In particular, the analysis of typical cell membrane markers as well as that of the release of pro-inflammatory agents, including myeloperoxidase enzyme activity for granulocytes and cytokines for monocytes/macrophages, were performed.

#### Granulocyte levels and myeloperoxidase (MPO) activity

PBS (10 µL) was used as a negative control, while the TSP-1 peptide RFYVVMWK (10 $\mu$L, 200$\mu$M in PBS) was used as a positive control. NP was added to flow cytometer vials, to which 100 µL of whole blood from individual donors was added and incubated, at room temperature, for 30 min. Samples were fixed by the addition of 110 µL of 0.4% (*v*/*v*) formaldehyde solution for 30 min. Samples were washed by the addition of 1 mL of PBS followed by centrifugation for 10 min at 500 × *g*. The supernatants were discarded, leaving approximately 100 µL volume, including the cell pellet. Anti-myeloperoxidase PE-labeled antibodies (5 μL, Becton Dickinson) were added to each sample and incubated for 1 h at room temperature and washed twice. Lysing solution (1 mL) was added for 10 min at room temperature. PBS (2 mL) was added, and samples were centrifuged at 500 × *g* for 10 min. The supernatants were aspirated and the cell pellets suspended in 500 µL of PBS prior to analysis by flow cytometry (Accuri C6, BD, UK).

### Monocyte/macrophage levels and cytokine release

For each 100 μL of whole blood required, 5 µl of FITC-labeled mouse anti-human CD14 (Becton Dickinson) and 5 µL of PE-labeled anti-human CD11b antibody (BioLegend) were added. Aliquots of 10 µL of either NP or TSP-1 peptide (positive control) or PBS (negative control) were added to 100 µL of antibody-labeled whole blood and incubated for 30 min, room temperature. Samples were fixed by the addition of 110 µL of 0.4% (*v/v*) formaldehyde solution (Sigma-Aldrich) to the cultures for 30 min. Samples were washed by the addition of 1 mL of PBS followed by centrifugation for 10 min at 500 × *g*. The supernatants were discarded, leaving approximately100 µL volume, including the cell pellet. Lysing solution (1 mL, Beckman Coulter) was added for 10 min at room temperature. Two washes in PBS (2 mL) were performed. The supernatants were aspirated and the cell pellets suspended in 500 µL of PBS prior to analysis by flow cytometry (Accuri C6, BD, UK).

Human Th1/Th2 cytokine standards (Cytometric Bead Array, CBA, BD, UK) were reconstituted in assay diluent and incubated for 15 min at room temperature (0–2500 pg/mL). After incubation, 6 human Th1/Th2 cytokine capture beads were treated with 50 µL PE-conjugated detection antibodies (BD, UK) before being mixed with 10 µL plasma/standards diluted in 40 µL of serum enhancement (BD, UK). Both samples and standards were then incubated for 3 h. After 3 washing steps with 1 mL of washing buffer, samples and standards were assessed by BD Accuri C6 flow cytometer, and results were analyzed using a specific BD analysis software. Data of blood samples from individual donors after spiking with different concentrations of NP were assessed for relative levels of cytokines compared to the control.

### Statistical analysis

Statistical analysis was performed by ANOVA *t*-test (Tukey Kramer test) from *n* = 6 blood donors. Significant differences of samples treated with different concentrations of NP from those of each donor’s control were tested at *p* < 0.05.

## Results and discussion

**Fig. 1:**
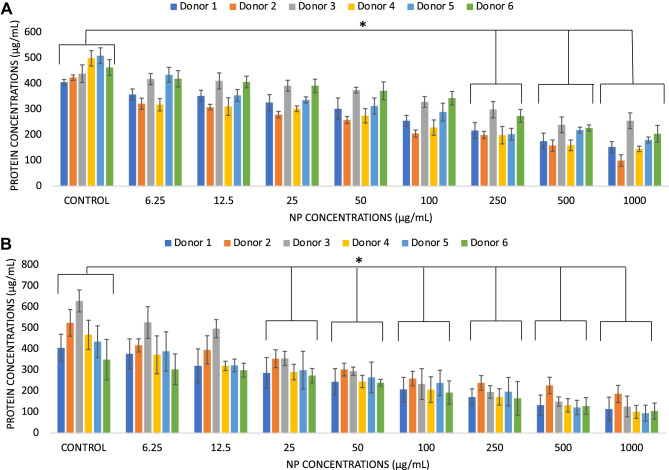
Levels of protein concentrations in human donors’ peripheral blood incubated with increasing concentrations of PEBCA **A** and LipImage™ 815 **B** NP. Protein levels are expressed as mean $\mu$g/mL from *n* = 6 donors, each tested in duplicate. Data are shown as mean + standard deviation from *n* = 3 replicates for each donor. * indicates *p* < 0.05

The measurement of the protein levels in the blood samples exposed to different concentrations of PEBCA and LipImage^TM^ 815 was adopted as indirect testing of the early interactions of the NP with blood components. Protein levels in freshly isolated whole heparinized human peripheral blood showed a statistically significant reduction of protein levels in all donors when relatively high concentrations (≥ 100$\mu$g/mL) of both PEBCA and LipImage^TM^ 815 were tested (Fig. 1A, B, individual donors’ data, Suppl. Fig. 1, pooled donors’ data, *p* < 0.05). These decreased levels indicated possible adsorption of blood proteins onto the NP surface or the possible precipitation of proteins induced by high concentrations of NP. The incubation time was chosen to evaluate both early protein adsorption and possible precipitation events. Indeed, while protein interactions with biomaterial surfaces are known to take place in milliseconds after the exposure of biomaterial surfaces to body fluids [18], over a longer period of time, the so-called Vroman’s effect takes place, whereby proteins with a relatively high molecular weight displace those with low molecular weight, leading to the formation of a stable protein layer [18]. In addition, serum protein depletion by NP incubation has been previously demonstrated, particularly in the case of NP with a negatively charged surface [19].

The consequent adsorption of this protein film at the biomaterial surface is also considered the main cause of the host response to implants, including thrombogenicity and inflammatory response [3]. Among the proteins relevant to the host response and able to adsorb onto biomaterial surfaces, fibrinogen plays a key role in the clot activation upon NP systemic administration [20].

**Fig. 2:**
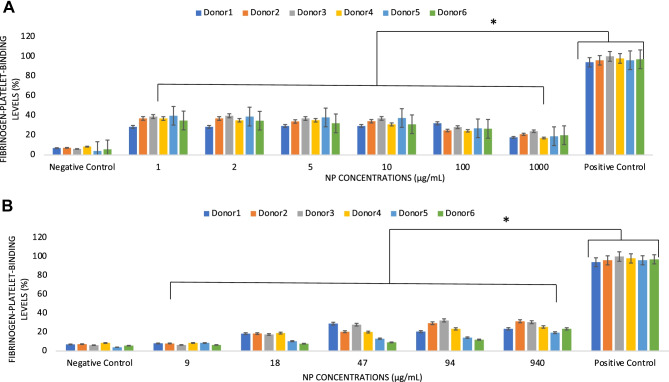
Levels of fibrinogen-platelet binding in human donors’ peripheral blood incubated with increasing concentrations of PEBCA **A** and LipImage™ 815 **B** NP. Data are expressed as mean ± standard deviation of the percentage of the positive control from *n* = 6 donors each tested in duplicate. Data are shown as mean + standard deviation from *n* = 2 replicates for each donor. * indicates *p* < 0.05

The data showed that fibrinogen-platelet binding was dependent on the type of NP tested but not exceeding 40% of the positive control (Fig. 2A, B). In the case of PEBCA, a binding level of ca 40% was observed in all donors in the range of concentrations 1 to 100$\mu$g/mL (Fig. 2A individual donors’ data, Suppl. Fig. 2A, pooled donors’ data, *p* < 0.05). This binding significantly dropped to ca 20% at 1000 $\mu$g/mL, following a pattern similar to that observed for the protein concentration levels. It can therefore be speculated that, in the case of these polymeric NP, the binding of platelets is limited and possibly reduced by the reduced concentrations of adsorbed fibrinogen. A different behavior was observed when different concentrations of LipImage^TM^ 815 NP were studied (Fig. 2B, individual donors’ data, Suppl. Figure 2B, pooled donors’ data, *p* < 0.05). This lipid-based nanocarrier showed a slight increase of fibrinogen-platelet binding at increasing NP concentrations but never exceeding the 30% level. In addition, higher individual variability was observed, with two out of 6 donors showing significantly lower levels of binding up to concentrations of 100 $\mu$g/mL. These observations are supported by previous studies that have demonstrated that the binding of fibrinogen sequences to liposomes induces the binding and activation of platelets [25, 26].

Fibrinogen binding to NP with different physicochemical properties has been widely documented and related to both the NP surface electrostatic charges and size [21, 22]. In the present protocol, the assay adopted assessed the binding of fibrinogen to platelets by spiking freshly isolated PRP with labeled fibrinogen. The assay provides indications of (i) the NP-induced activation of platelets leading to a higher expression of their receptors for fibrinogen and consequently of thrombogenicity through fibrinogen-platelet complex formation, (ii) the binding of the formed fibrinogen to the surface of the NP that may also trigger thrombogenicity through complexation with the platelets. Platelet activation measured as an expression of CD63 and CD62b antibodies was negligible for both NP types until concentrations of 1000 micrograms/mL were tested, levels of ca 30% of the positive controls were observed (data not shown). Therefore, it is suggested that the adsorption of fibrinogen rather than direct activation of the platelets by NP may determine mild thrombogenicity potential. It may be speculated that the different profiles of fibrinogen-platelet binding on the two types of NP could be linked to their different physicochemical properties. The data suggest that the fibrinogen adsorbed within the PEBCA NP protein corona favors platelet binding at low NP concentrations and that only the alleged depletion of proteins observed at relatively high concentrations (> 100 $\mu$g/mL) slightly reduces this binding. It is also observed that in such a case, the presence of PEG surfactants in the PEBCA NP formulation did not prevent the adsorption of fibrinogen and consequent platelet binding. These findings are corroborated by previous studies showing that PEG reduces but does not completely prevent the formation of a protein corona and fibrinogen binding at the surface of NP and that it is rather changing its composition and it is the cause of serum protein depletion as that observed in the experiments of protein concentration [23–26]. In addition, the formation of a protein corona at the surface of NP has been linked to their longer stability in vivo [26]. Conversely, the intrinsically more unstable nature of the lipid-based LipImage^TM^ 815 appears to induce a reduced and more variable formation of the fibrinogen-platelet complex [26–28].

In the proposed NP testing protocol, the combination of the simple and relatively rapid Bradford’s protein concentration assay and the flow cytometry study of fibrinogen-platelet binding highlights the different hemocompatibility of NP under investigation, particularly in relation to the early interaction of these NP with blood components following systemic administration.

**Fig. 3:**
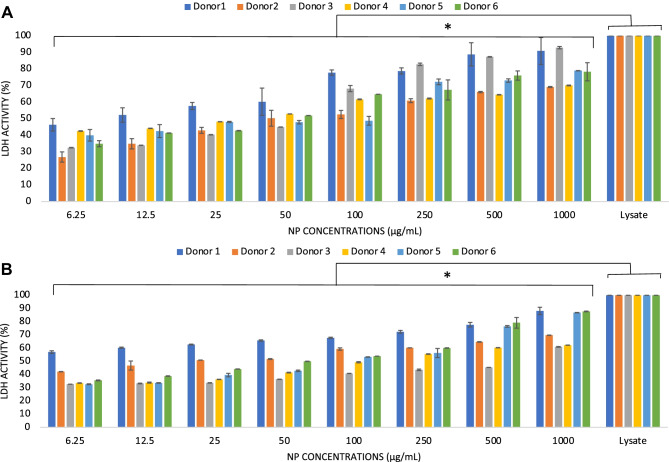
Levels of cytotoxicity in human blood induced by increasing concentrations of PEBCA **A** and LipImage™ 815 **B** NP. Release LDH activity is expressed as mean ± standard deviation of the percentage of the positive control from *n* = 6 donors each tested in duplicate. Data are shown as mean + standard deviation from *n* = 2 replicates for each donor. * indicates *p* < 0.05

**Fig. 4:**
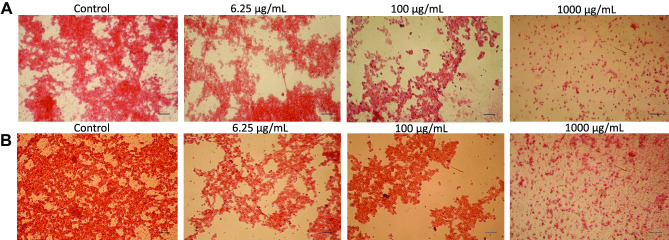
Cytotoxicity of deposited human blood cells induced by increasing concentrations of PEBCA (panel **A**) and LipImage™ 815 (panel **B**) NP. Cells were stained by HE method, and photos were taken by light microscopy at × 20 magnification. Images were taken at × 10 magnification

The availability of relatively large volumes (5 mL) of blood samples also enabled a further assessment of the host response to the tested NP in relation to their cytotoxicity and inflammatory response. To this end, aliquots of the same samples used for the protein concentration measurements were used to assess the NP cytotoxicity. Indeed, these samples provided the opportunity to test the loss of cell integrity by released LDH activity (Fig. 3A, B) in the blood volume and that of cells, mainly erythrocytes, deposited at the bottom of the well (Fig. 4A, B). The basal LDH activity of the tested blood donors, measured as absorbance at 490 nm, was 0.534 ± 0.044, while the average LDH activity released by induced cell lysis (positive control) was 1.651 ± 0.270. An increase in the LDH activity was observed only at concentrations of 100 $\mu$g/mL and above for both NP types (Fig. 3A, B) with clear individual donor variations. A direct comparison between the two types of NP was obtained by an analysis of the donors’ pooled data showing similar cytotoxic levels for the two nanocarriers (Suppl. Fig. 3). The loss of cell integrity was qualitatively confirmed by HE staining of the well surface that showed reduced staining of deposited cells, mainly erythrocytes, at the same concentrations for both types of NP (Fig. 4 panels A and B) in line with previously published data of erythrocyte toxicity by NP [29].

**Fig. 5:**
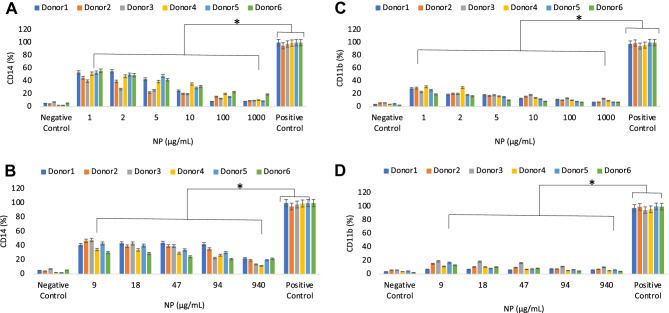
Expression of blood inflammatory cells’ membrane markers upon spiking with increasing concentrations of PEBCA **A**, **C** and LipImage™ 815 **B**, **D** NP. Flow cytometry data for CD14 **A** and **B** and CD11b **C** and **D** expression are expressed as mean ± standard deviation of the percentage of the positive control from *n* = 6 donors each tested in duplicate. Data are shown as mean + standard deviation from *n* = 2 replicates for each donor. * indicates *p* < 0.05

Whole human blood samples enabled the assessment of the inflammatory response to NP, another key parameter in the study of biomaterial hemocompatibility. This last batch of experiments of the multi-phase protocol was linked to the data of protein concentrations and cytotoxicity to indicate whether the incubation with the two types of NP was affecting the mononuclear cell population and its role in inducing an inflammatory response either by cell activation or by the unintended release of pro-inflammatory cytokines through loss of cell integrity. As the inflammatory response to biomaterials is triggered by the early activation of granulocytes followed by that of monocytes/macrophages [30, 31], the flow cytometry analysis of this study focused on the markers of these two types of cells as well as on the release of pro-inflammatory macromolecules. In this part of the protocol, the flow cytometry tests focused on the presence of cells in the human blood expressing CD14 and CD11b, cell surface markers common to both granulocytes and monocytes/macrophages [32, 33]. The levels of CD14 observed at increasing concentration of PEBCA NP showed a gradual reduction of the levels of these cells, suggesting that relatively high concentrations of NP, consistently with the data of cytotoxicity, led to the loss of cell integrity (Fig. 5A, Suppl. Fig. 4A, C). Similar results were observed for LipImage^TM^ 815 (Fig. 5B, Suppl. Fig. 4B, D).

It has been reported that under inflammatory conditions, CD11b (also known as Mac-1) expression is upregulated and differential CD11b can distinguish between the peak and resolution of acute lung inflammation, respectively. Mac-1 is expressed on monocytes, neutrophils, peritoneal B-1 cells, CD8^+^ dendritic cells, NK cells, and a subset of CD8^+^ T cells [33]. It functions in cellular adhesion, phagocytosis, and extravasation, and it may play a role in chemotaxis. Mac-1 binds a diverse group of ligands, which include inactivated complement component C3b (iC3b), fibrinogen, coagulant factor X, and the intercellular adhesion molecule ICAM-1. In this work, CD11b expression induced by the tested NP never exceeded 20% of the positive control, and a trend toward its reduction at higher concentrations was related to the demonstrated loss of blood cell integrity (Fig. 5C, D). Hence, the combination of the CD14 and CD11b data seem to indicate relatively low levels of inflammatory cells’ activation by the tested NP.

**Fig. 6:**
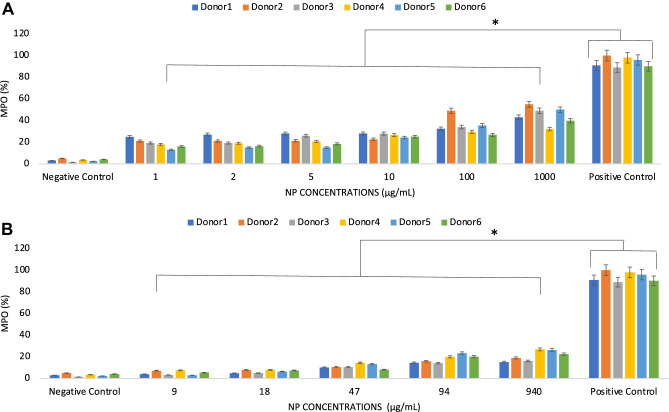
Levels of MPO activity released by granulocytes in human blood spiked by increasing concentrations of PEBCA **A** and LipImage™ 815 **B** NP. Flow cytometry data are expressed as mean ± standard deviation of the percentage of the positive control from *n* = 6 donors each tested in duplicate. Data are shown as mean + standard deviation from *n* = 2 replicates for each donor. * indicates *p* < 0.05

However, the study of the release of soluble pro-inflammatory pathways seemed to suggest that an inflammatory insult could derive from the cell death detected at the relatively high NP concentrations. Indeed, granulocyte MPO activity [34] increased with the increase of NP concentrations, with a higher release being observed in the case of PEBCA NP, where the MPO activity ranged from approximately 20 to 50% of the positive control (Fig. 6A, Suppl. Fig. 5A). In the case of LipImage^TM^ 815, MPO activity was relatively low reaching a maximum of approximately 20% at the highest concentrations tested and being not significantly different from the negative control up to 50 $\mu$g/mL (Fig. 6B, Suppl. Fig. 5B).

For all these markers of the inflammatory response, the reproducibility of the data across donors is highlighted when donors’ data were pooled, showing a significantly (*p* < 0.05) lower activation of the inflammatory cells when compared to the positive controls (Suppl. Fig. 4A–D and Suppl. Fig. 5A, B).

**Fig. 7:**
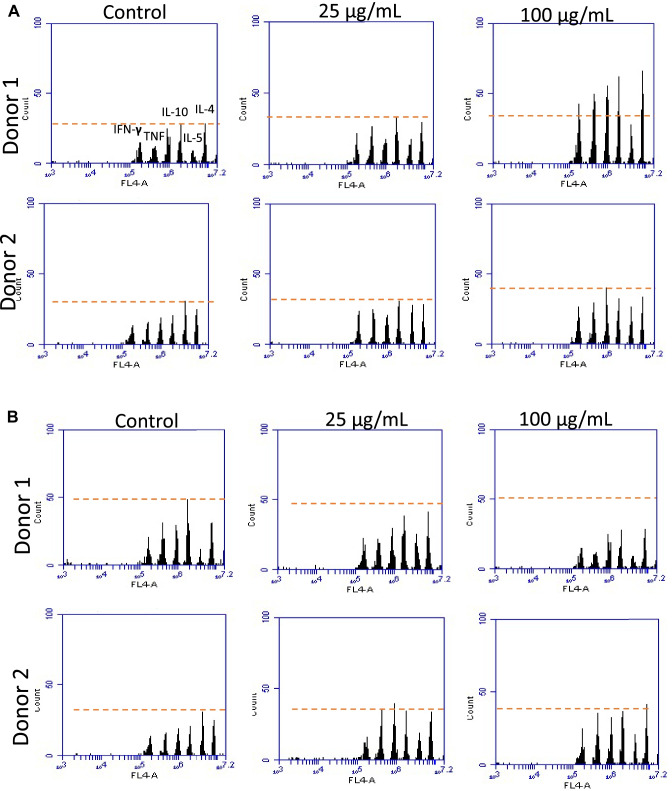
Semi-quantitative assessment of the levels of cytokine released inflammatory cells (granulocytes and mononuclear cells) in human blood spiked by increasing concentrations of PEBCA (Panel A) and LipImage^TM ^815 (Panel B). Flow cytometry plot of control sample from Donor n. 1 reports the Th1/Th2 signalling markers analysed in the assay; $\mathrm{IFN}\gamma$, TNF, IL-10, IL-5, IL-4. Flow cytometry plots are representative of donors showing comparable or different release NP response profiles to those of those of the relative control.

Finally, flow cytometry was applied to test the Th1/Th2 pathway that is shared by most of the blood immunocompetent cells (i.e., monocytes/macrophages and lymphocytes) [35], thus enabling us evaluate the immune response to NP more comprehensively. The flow cytometry analysis was performed by commercially available antibody-functionalized microbead arrays to study the release of other relevant pro-inflammatory factors at different NP concentrations (Fig. 7, panels A and B). The flow cytometry panels for both PEBCA NP and LipImage^TM^ 815 NP showed release of the tested cytokines at levels not significantly different from the negative control (blood samples not challenged by NP) when the tested NP concentrations were below the determined cytotoxic threshold. Only a slight and donor’s dependent higher release of some of the tested cytokines was observed at the cytotoxic concentrations. As observed in the case of other assays of the multistep protocol, the testing of blood samples from individual donors highlighted that the levels of release of the cytokines of the Th1/Th2 pathway showed, once again, the importance of assessing NP hemocompatibility in the contest of individual variability.

Taken together, the data obtained by this multistep protocol demonstrates its ability to discriminate between different components of the host response, different doses, and different NP physicochemical properties, as well as individual variability. The comparable size of the NP and the same range of concentration tested in this study also corroborate the comparative value offered by the protocol. The tested NP were not showing a significant activation of the host response in most of its measured parameters. The thrombogenic potential seemed to be affected slightly by both the type of NP tested and their ability to induce blood protein depletion. At the same time, while pronounced lysis of erythrocytes was observed, the integrity of the remaining blood cell population seemed to be compromised only when very high NP concentrations were tested, resulting in low levels of release of pro-inflammatory cytokines.

## Conclusions

Future developments and regulatory approval of nanoparticulate carriers will depend on the definition of reliable testing protocols able to identify in a user-friendly, cost-effective, and relatively fast manner the host response triggered upon administration. At the same time, it is argued that these tests should also provide reassurance about the variability of the host response of individual patients. The present work shows that a multistep protocol obtained from single collections of peripheral blood from different donors may provide a first level of safety check of NP with different physicochemical properties during their phase of product development and provide batches of in vitro data suitable for regulatory approval. In addition, this sequence of tests could be adopted to predict any adverse effect occurring on single patients and help to establish personalized safety assessments and dose regimes of nanomedicines.

## Supplementary information

Below is the link to the electronic supplementary material.

Supplementary file1 (TIF 518 KB). Supplementary Figure 1. Pooled data of the levelsof protein concentrations in human donors’ peripheral blood incubated with increasing concentrations of PEBCA andLipImage^TM^ 815 NP. Data are expressed as mean micrograms/mL from n=6 donors eachtested in duplicate. Statistical annotations: * p <0.05.

Supplementary file 2 (TIF ). Supplementary Figure 2. Pooled data of fibrinogen-platelet binding in human donors' peripheral blood incubated with increasing concentrations of PEBCA (A) and LipImage^TM^ 815 (B) NP. Data are expressed as mean +/- standard deviation of the percentage of the positive control from n=6 donors each tested in duplicate. * indicates p <0.05.

Supplementary file 3 (TIF 552 KB). Supplementary Figure 3. Pooled data of levels of cytotoxicity in human blood induced by increasing concentrations of PEBCA andLipImage^TM^ 815 NP. Released LDH activity is expressed as mean +/- standard deviation of the percentage of the positive control from n=6 donors each tested in duplicate. * indicates p <0.05.

Supplementary file 4 (TIF 523 KB). Supplementary Figure 4. Pooled data of expression of blood inflammatory cells’ membrane markers upon spiking with increasing concentrations of PEBCA (A, C) and LipImage^TM^815 (B, D) NP. Flow cytometry data for CD14 (A and B) and CD11b (C and D) expression are expressed as mean + standard deviation of the percentageof the positive control from n=6 donors each tested in duplicate. * indicates p<0.05.

Supplementary file 5 (TIF 634 KB). Supplementary Figure 5. Pooled data of levels of MPO activity released by granulocytes in human blood spiked by increasing concentrations of PEBCA (A) and LipImage^TM^ 815 (B) NP. Flow cytometry data are expressed as mean +/- standard deviation of the percentage of the positive control from n=6 donors each tested in duplicate. *indicates p <0.05.

## Data Availability

Raw data are stored in the University of Brighton repository and made available upon request.
