# Supplementary figures and images for: A multistep in vitro hemocompatibility testing protocol recapitulating the foreign body reaction to nanocarriers
Source: Drug Deliv Transl Res. 2022 Mar 22;12(9):2089–100. doi: 10.1007/s13346-022-01141-6 (PMC9360154; doi:10.1007/s13346-022-01141-6)

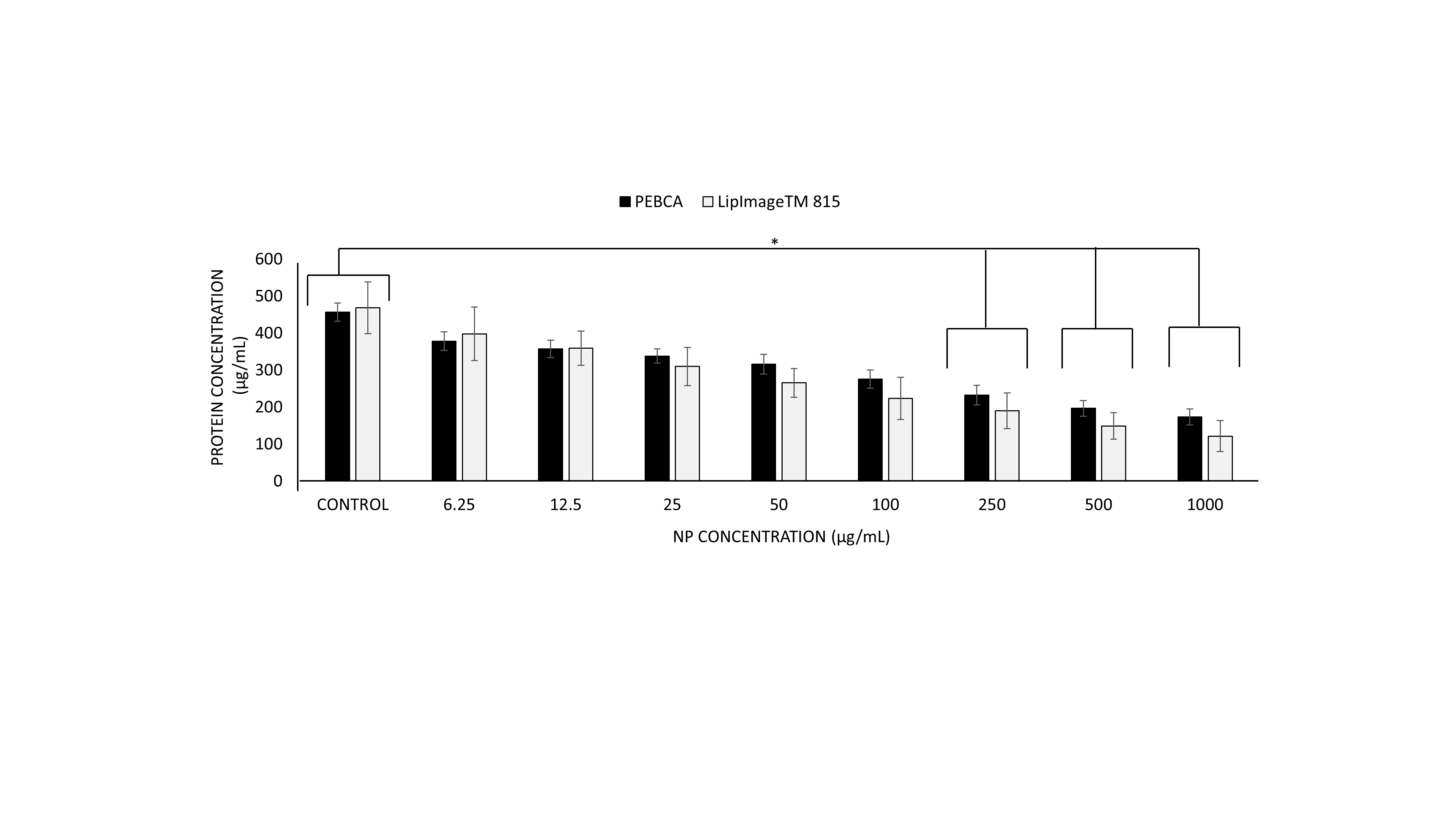

Supplement: Supplementary file 1 — Supplementary file1 (TIF 518 KB). Supplementary Figure 1. Pooled data of the levelsof protein concentrations in human donors’ peripheral blood incubated with increasing concentrations of PEBCA andLipImageTM 815 NP. Data are expressed as mean micrograms/mL from n=6 donors eachtested in duplicate. Statistical annotations: * p <0.05. [file 13346_2022_1141_MOESM1_ESM.tif]

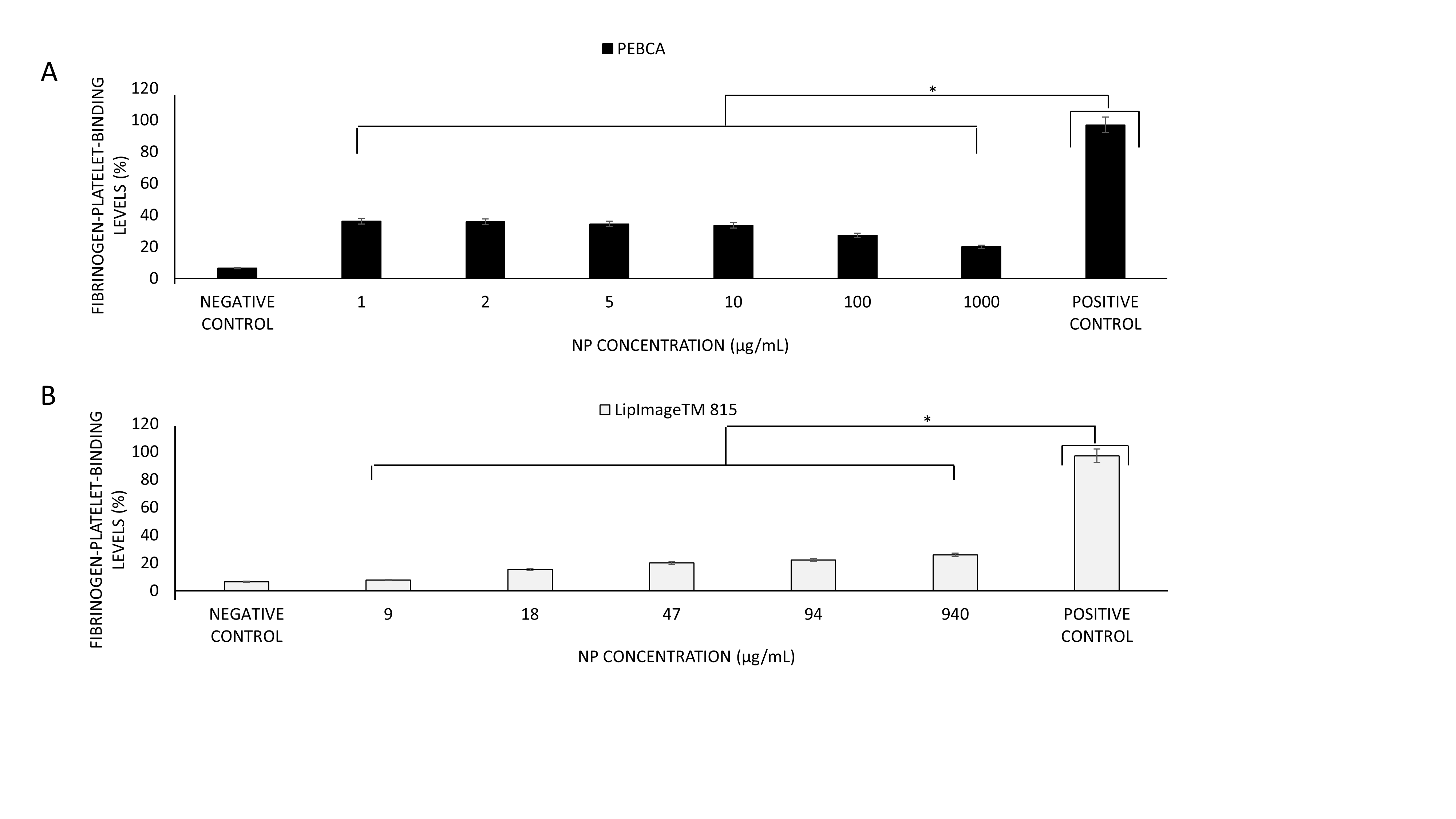

Supplement: Supplementary file 2 — (TIF ). Supplementary Figure 2. Pooled data of fibrinogen-platelet binding in human donors' peripheral blood incubated with increasing concentrations of PEBCA (A) and LipImageTM 815 (B) NP. Data are expressed as mean +/- standard deviation of the percentage of the positive control from n=6 donors each tested in duplicate. * indicates p <0.05. [file 13346_2022_1141_MOESM2_ESM.tif]

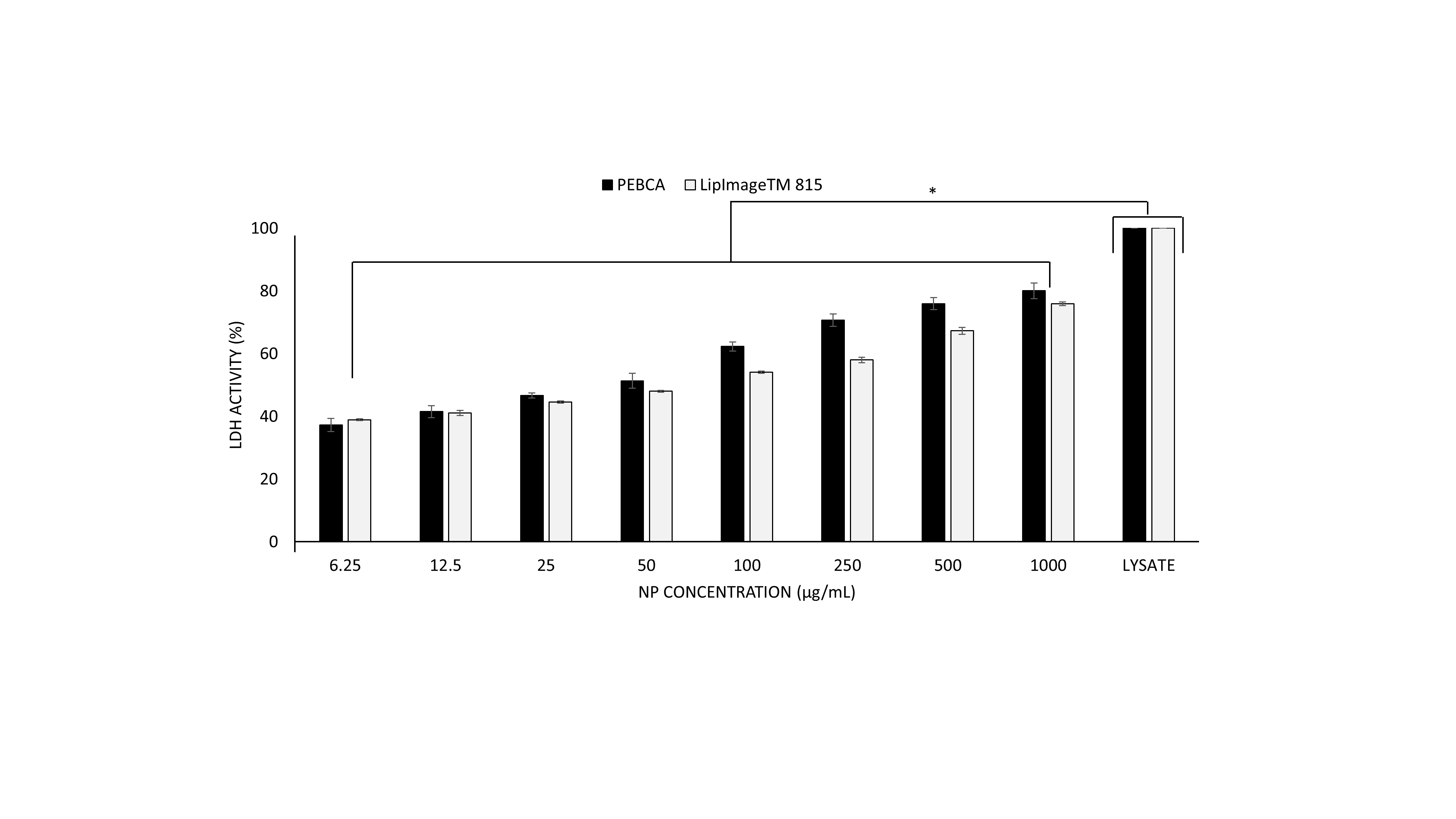

Supplement: Supplementary file 3 — (TIF 552 KB). Supplementary Figure 3. Pooled data of levels of cytotoxicity in human blood induced by increasing concentrations of PEBCA andLipImageTM 815 NP. Released LDH activity is expressed as mean +/- standard deviation of the percentage of the positive control from n=6 donors each tested in duplicate. * indicates p <0.05. [file 13346_2022_1141_MOESM3_ESM.tif]

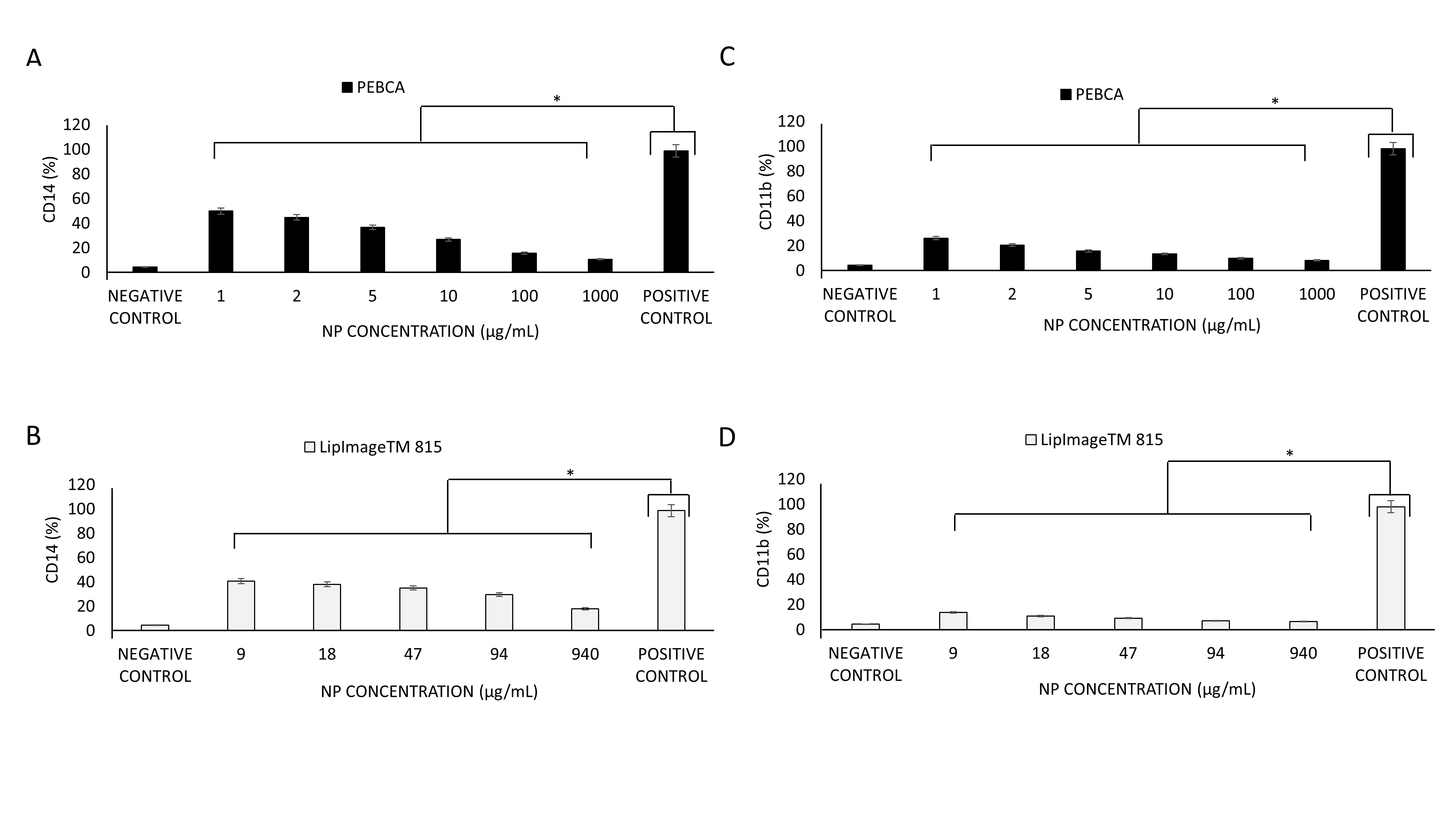

Supplement: Supplementary file 4 — (TIF 523 KB). Supplementary Figure 4. Pooled data of expression of blood inflammatory cells’ membrane markers upon spiking with increasing concentrations of PEBCA (A, C) and LipImageTM815 (B, D) NP. Flow cytometry data for CD14 (A and B) and CD11b (C and D) expression are expressed as mean + standard deviation of the percentageof the positive control from n=6 donors each tested in duplicate. * indicates p<0.05. [file 13346_2022_1141_MOESM4_ESM.tif]

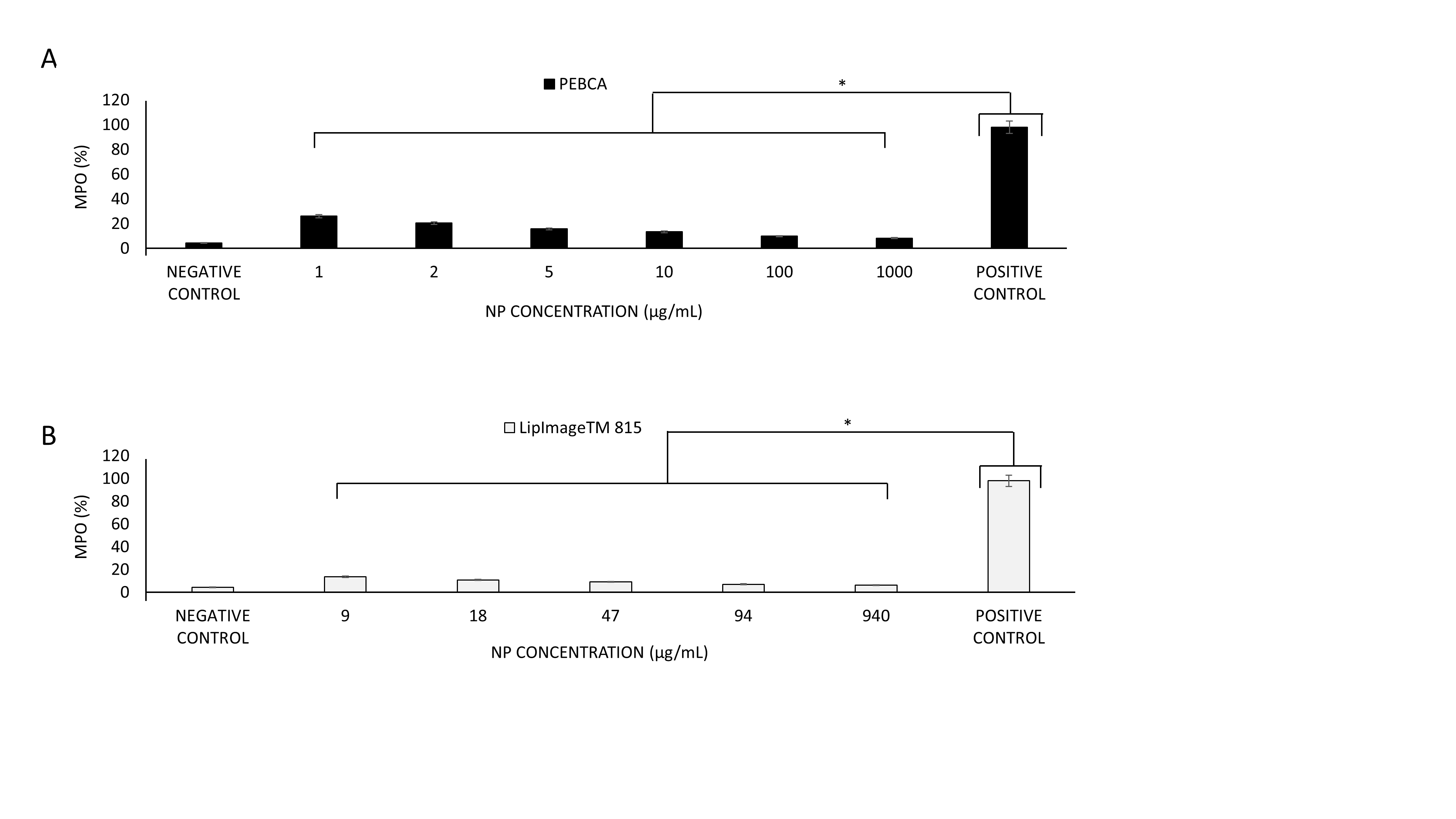

Supplement: Supplementary file 5 — (TIF 634 KB). Supplementary Figure 5. Pooled data of levels of MPO activity released by granulocytes in human blood spiked by increasing concentrations of PEBCA (A) and LipImageTM 815 (B) NP. Flow cytometry data are expressed as mean +/- standard deviation of the percentage of the positive control from n=6 donors each tested in duplicate. *indicates p <0.05. [file 13346_2022_1141_MOESM5_ESM.tif]
